# The effects of flaxseed supplementation on metabolic status in women with polycystic ovary syndrome: a randomized open-labeled controlled clinical trial

**DOI:** 10.1186/s12937-020-0524-5

**Published:** 2020-01-24

**Authors:** Fatemeh Haidari, Nasrin Banaei-Jahromi, Mehrnoosh Zakerkish, Kambiz Ahmadi

**Affiliations:** 1 Department of Nutrition, Nutrition and Metabolic Diseases Research Center, Faculty of Paramedical Sciences, Ahvaz Jundishapur University of Medical Sciences, Ahvaz, 61357-15794 Iran; 2 Department of Endocrinology and Metabolism, Faculty of Medicine, Diabetes Research Center, Ahvaz Jundishapur University of Medical Sciences, Ahvaz, Iran; 3 Department of Statistics and Epidemiology, Faculty of Public Health, Ahvaz Jundishapur University of Medical Sciences, Ahvaz, Iran

**Keywords:** Polycystic ovary syndrome, Flaxseed, Sex hormone binding globulin, Insulin resistance, Adiponectin, Leptin

## Abstract

**Background:**

Polycystic Ovary Syndrome (PCOS) is known as the most common endocrine disorder of women in reproductive ages. With the increasing prevalence of PCOS in different countries, the use of herbal medicine as an alternative treatment is growing in these patients. This study aimed to evaluate the effects of flaxseed powder supplementation on metabolic biomarkers of patients with PCOS.

**Methods:**

This randomized open-labeled controlled clinical trial was conducted on 41 patients with PCOS. The participants were randomized to take either flaxseed powder (30 g/day) plus lifestyle modification or only lifestyle modification for 12 weeks. Anthropometric and biochemical evaluations were performed for all patients at the beginning and end of the study.

**Results:**

The flaxseed group showed a significant reduction in body weight, insulin concentration, Homeostatic Model Assessment of Insulin Resistance (HOMA-IR), Triglycerides (TG), high-sensitivity C-Reactive Protein (hs-CRP), and leptin and an increase in Quantitative Insulin-Sensitivity Check Index (QUICKI), High Density Lipoprotein (HDL), and adiponectin compared to the baseline (*p* < 0.05). Flaxseed supplementation also led to a significant reduction in insulin concentration, HOMA-IR, TG, hs-CRP, Interleukin 6 (IL- 6), and leptin and an increase in QUICKI, HDL, and adiponectin compared to the control group (*p* < 0.05). No significant changes were observed in other parameters.

**Conclusions:**

Flaxseed supplementation plus lifestyle modification was more effective compared to lifestyle modification alone in biochemical and anthropometric variables in patients with PCOS.

**Trial registration:**

The trial protocol was approved by the Ethics Board at Ahvaz Jundishapur University of Medical Sciences and was registered at Iranian Registry of Clinical Trials (code: IRCT20120704010181N11).

## Background

Polycystic Ovary Syndrome (PCOS) is known as the most common endocrine disorder of women in reproductive ages, with the prevalence ranging from 6 to 15% [1, 2]. The most common clinical manifestations of this syndrome include irregular menstrual cycles, infertility, acne, hair loss with the male pattern, Insulin Resistance (IR), and obesity [3, 4]. According to Rotterdam criteria, PCOS diagnosis depends on the identification of at least two of the following three features: oligo-anovulation, hyperandrogenism, and polycystic ovaries in ultrasound [5].

Patients with PCOS are at increased risk for the development of metabolic syndrome, type II diabetes mellitus, and Cardiovascular Disease (CVD) [1, 6–8]. Although the main mechanism leading to PCOS remains unknown, obesity and IR seem to play a key role in the pathogenesis of this syndrome [9]. Obesity leads to more than 50% IR in these patients and is associated with different metabolic abnormalities, especially increased androgen production [10]. Hyperinsulinemia caused by IR leads to hyperandrogenemia through excess production of androgens by Theca cells in ovaries and also reduces the liver synthesis of Sex Hormone-Binding Globulin (SHBG) [6, 8]. It is well known that the adipose tissue, specifically visceral fat, is associated with IR, diabetes, hypertension, and pro-inflammatory states [2]. Adipose tissue is now considered to be a secretory organ for adipocytokines, such as adiponectin and leptin, which are involved in the PCOS pathogenesis [11].

Lifestyle modifications, such as dietary pattern, exercise, and behavioral therapies, are the first line of treatment for PCOS [12]. Yet, attention to medicinal herbs has recently expanded as an alternative treatment or diseases control [13].

Flaxseed (*Linum usitatissiumum*) is a rich source of several biologically active compounds, including α-Linolenic Acid (ALA), phytosterogenic lignans (secoisolariciresinol diglycoside-SDG), and dietary fibers [14, 15]. Prior studies have indicated that high lignin foods increased testosterone exertion by binding it to enterohepatic circulation [16]. Lignans could also reduce the bioavailability of free testosterone through increasing SHBG levels [17]. To the best of our knowledge, there are scarce data regarding the effects of flaxseed powder supplementation on lipid profile, insulin sensitivity, inflammatory markers, leptin, adiponectin, and menstrual irregularities in patients with PCOS. Therefore, the present study aims to assess the effects of flaxseed powder on metabolic and anthropometric statuses in these patients.

## Methods

### Trial design and participants

This study was a randomized open labeled controlled clinical trial with two parallel groups that was performed from May to November 2018. Patients were recruited from the endocrinology clinic of Golestan Hospital, Ahvaz, Iran. The trial protocol was approved by the Ethics Board at Ahvaz Jundishapur University of Medical Sciences and was registered at Iranian Registry of Clinical Trials (code: IRCT20120704010181N11). Indeed, the researchers adhered to the Consolidated Standards of Reporting Trials (CONSORT) guidelines for reporting on randomized clinical trials (See Supplementary Materials) [18]. Written informed consent forms were also obtained before the participants were enrolled into the study.

PCOS was diagnosed based on the Rotterdam criteria [19]. The patients who were willing to participate in the trial were evaluated by a gynecologist and were included in the study if they met the Rotterdam criteria. Totally, 48 patients within the age range of 18–45 years were eligible if they were not menopause, did not consumed omega-3 in the last 3 months or followed a special diet, did not used tobaccos, and did not have diabetes or hypothyroidism. Patients were excluded in case of (1) a history of allergy and high consumption of nuts, flaxseed, or sesame seeds, (2) pregnancy or lactation, (3) hyperandrogenism caused by Cushing syndrome or androgen-secreting tumor, (4) hyperprolactinemia, (5) thyroid dysfunction and (6) if they consuming contraceptive.

### Study design

The participants were randomly allocated into two groups to take either 30 g/day brown milled flaxseed (*n* = 24) together with Lifestyle Modification (LM) or LM alone (n = 24) for 12 weeks. According to the study conducted by Hutchins et al. (2013) on Homeostatic Model Assessment of Insulin Resistance (HOMA-IR) and considering the confidence interval of 95% and power of 80%, the sample size was estimated to be 22 participants in each group. However, considering a 10% probable withdrawal, 24 participants were enrolled into each group. Randomization was performed by using the table of random numbers. Randomization and allocation concealment were conducted by the researchers and participants and were carried out by a trained staff at the gynecology clinic.

Flaxseed of wholesale was purchased and preserved in a cool and dark place before the onset of the study. It was milled and packed in 30 g packets one week before delivery to the patients. The patients were also asked to keep the flaxseed powder in a cool place. The dose of 30 g/day for flaxseed powder was chosen based on the recommendations from three systematic reviews and meta-analyses, which suggested that 30 g/day flaxseed powder or greater doses were most effective in normalizing inflammatory biomarkers, lipid profiles, and body composition for up to 12 weeks [20, 21]. Nonetheless, flaxseed powder in doses greater than 50 g/day should be used with caution to prevent side effects [22]. Nutrient content per serving (100 g) of the flaxseed powder was evaluated by a food laboratory in Ahvaz. The results were as follows: energy = 465 kcal, fat = 39 g, ALA = 21 g, protein = 18 g, carbohydrate = 29 g, and fiber = 28 g. Follow-up evaluations were performed every three weeks at weeks 3, 6, 9, and 12.

At the first visit (week 0), a checklist was completed for each participant and baseline data such as the type of medication, symptoms of hyperandrogenism, weight and waist circumference (WC) were fulfilled. Also, patients in the intervention group were given a three week supply of milled flaxseed. The participants were asked to add flaxseed to their salad, yogurt, or cold drinks and consume them during the day. Both groups received healthy nutritional recommendations based on the American Heart Association [23]. Indeed, they were asked to gradually increase their physical activity (> 30 min of moderate intensity physical activity three times per week) and inform to the research team if medications are changed.

### Compliance

In the third, sixth and ninth weeks, the participants were asked to come to the clinic and take their flaxseed powders. To enhance compliance with the study, short messages were sent to all flaxseed group participants’ cell phones every day to remind them about taking the flaxseed powder. Biochemical, anthropometric and nutritional factors were evaluated only at the beginning and end of the study. The degree of compliance for each patient was determined according to the amount of the returned flaxseed powder. In case they had consumed less than 90% of the prescribed flaxseed powder, they were excluded from the analysis.

### Assessment of dietary intake and physical activity

To evaluate dietary intake, all patients completed a 3-day 24-h dietary recall at baseline and at the end of the study. Daily macro-and micro-nutrients intakes were analyzed by nutritionist IV software (First Databank, San Bruno, CA). The short form of the International Physical Activity Questionnaire (IPAQ) was used to assess the level of physical activity at the beginning and end of the study, which was defined by Metabolic Equivalents (METs) in min/week [24].

### Assessment of anthropometric measures

Anthropometric indices were measured at baseline and at the end of the study. Body weight was measured in the fasting state with light clothing and without shoes using a digital scale (Seca, Hamburg, Germany). Height was also measured without shoes using an inelastic measuring tape. Body Mass Index (BMI) was calculated using the standard formula as follows: weight (kg) / height (m^2^). Finally, waist circumference was obtained according to World Health Organization’s (WHO) recommendation using a non-stretchable measuring tape [25]. To measure the effect of the menstrual cycle on weight and waist circumference changes, we performed these measurements both at the beginning and at the end of the study on days other than the menstrual cycle.

### Assessment of outcomes

Insulin metabolism markers [serum insulin level, HOMA-IR, and Quantitative Insulin-Sensitivity Check Index (QUICKI)], androgenic indices [total testosterone, SHBG, and Free Androgen Index (FAI)], and clinical variables [hirsutism and menstrual cycles status] were considered as the primary outcomes. Other biomarkers, such as serum levels of lipid profiles, fasting blood glucose, inflammation markers, and adipokines, were considered as the secondary outcome variables.

Clinical evaluations included determination of hirsutism using *Modified* Ferriman–Gallwey (mFG) score [26] and assessment of menstrual cycles regularity using personal interviews. At the beginning and end of the study, all patients were interviewed by the research team to determine whether the menstrual cycle was regular or not.

### Biochemical analysis

Blood samples (10 mL) were collected early in the morning after an overnight fasting at the beginning and end of the trial. Serum samples were separated from the whole blood by centrifugation at 2606.8×g for 10 min. Serum samples were stored at − 80 °C until laboratory analyses. Serum glucose, Total Cholesterol (TC), High-Density Lipoprotein Cholesterol (HDL-C), and Triglycerides (TG) concentrations were measured via the standard enzymatic method using Pars Azmoon kits (Pars Azmoon Inc., Tehran, Iran). Inter- and intra-assay Coefficient of Variation (CV) for fasting blood glucose and lipid concentrations were less than 5%. Low-Density Lipoprotein Cholesterol (LDL-C) was calculated by the Friede-Wald formula: LDL-C (mg/dl) = TC - (HDL – C + TG/5) [27].

Serum insulin concentration was determined by using ELISA kits (Dia plus, Canada) with inter- and intra-assay CVs of less than 12% and less than 10%, respectively. IR was determined by HOMA-IR using the following formula: HOMA-IR = [fasting insulin (mU/L) × fasting blood glucose (mg/dL)] / 405 [1]. Insulin sensitivity was also calculated by QUICKI equation: QUICKI = 1 / [log (fasting insulin, lU/mL) + log (fasting glucose, mg/dL)] [1]. Serum total testosterone with inter- and intra-assay CVs of 9.7 and 4.8% was determined by using direct immunoenzymatic assay (Accu Bind, USA). Besides, SHBG concentrations were assessed using ELISA Reader (IBL, USA). Serum hs-CRP and IL-6 inter- and intra-assay CVs of less than 12% and less than 10%, respectively were determined by commercial ELISA kits (DBC, Canada and EASTBIOPHARM, PRC). Additionally, FAI was calculated as the ratio of total testosterone to SHBG. Serum concentrations of adiponectin and leptin were also measured with inter- and intra-assay inter- and intra-assay CVs of less than 12% and less than 10%, respectively inter- and intra-assay CVs of less than 12% and less than 10%, respectively using ELISA kits (EASTBIOPHARM, PRC, and DBC, Canada). All assays were performed following the manufacturer’s instructions for each kit.

### Statistical analysis

The normality of all variables was checked using Kolmogorov-Smirnov test. For normal distribution variables, independent sample t-test and paired sample t-test were used to compare parameters at the beginning and at the end of the study between and within groups, respectively. Mann–Whitney U test and Wilcoxon signed-rank test as nonparametric alternatives were applied to compare sample parameters between and within groups, respectively. Patients’ medications were compared between the two groups was evaluated by chi-square test. To control confounding variables, including baseline values, age, baseline BMI, physical activity and baseline calorie of diet, Analysis of Covariance (ANCOVA) was used. The data were expressed as mean ± Standard Deviation (SD). All statistical analyses were performed using the SPSS 24 software, and *p* < 0.05 was considered to be statistically significant.

## Results

**Fig. 1:**
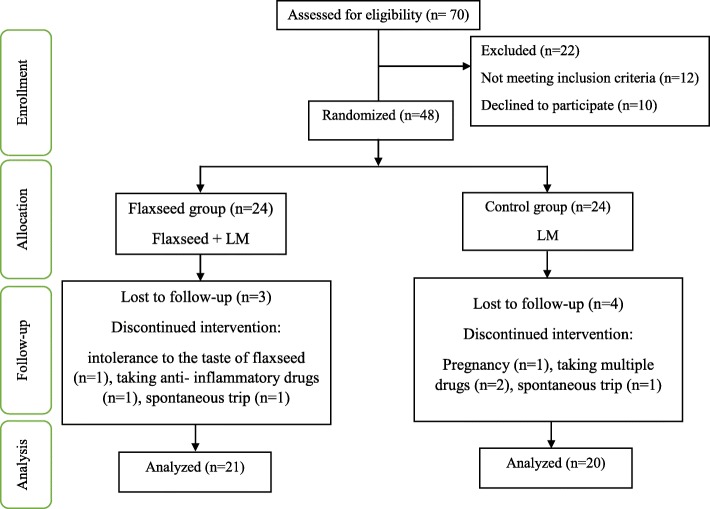
Flow chart of patient recruitment for the clinical trial of flaxseed supplementation in polycystic ovary syndrome

In the present study, 41 (85%) participants completed the trial. Seven participants (three in the flaxseed group and four in the control group) withdrew from the study due to personal reasons, migration, trip, pregnancy, and failure to follow the protocol (Fig. 1). The participants in the intervention group consumed more than 90% of flaxseed powder packs. Some patients taking flaxseed powder complained about bloating, abdominal pains, or sour throat. The mean age of the patients who finished the study in the two groups was 26.68 ± 6.11 years.

Baseline characteristics of the participants have been shown in Table 1. Accordingly, there was no significant difference between the two groups regarding mean age, weight, BMI, waist circumference, and physical activity level at baseline (*P* > 0.05). We evaluated the medications used by the patients using chi-square test and no significant differences were observed between the two groups (*P* > 0.05). Also, based on the 3-day dietary recalls at baseline and end of the study, there was no significant difference between the two groups concerning dietary components intake (*P* > 0.05) (Table 2).

**Table 1:** General characteristics in the intervention and control groups

Characteristics	Total (*n* = 41)	Flaxseed group (*n* = 21)	Control group (*n* = 20)	*P*-value^*^
Age (years)	26.68 ± 6.11	27.21 ± 5.89	26.13 ± 6.43	0.708
Weight (kg)	75.31 ± 15.92	73.90 ± 15.20	76.80 ± 16.86	0.547
WC (cm)	90.16 ± 12.49	88.71 ± 10.99	91.68 ± 13.99	0.433
BMI (kg/m2)	28.65 ± 5.64	28.38 ± 5.15	28.94 ± 5.63	0.966
METs (min/week) at study baseline	2543.53 ± 256.39	2540.47 ± 286.52	2546.75 ± 227.93	0.938
METs (min/week) at end	2551/21 ± 279/78	2568/80 ± 261/46	2534 ± 207/91	0.639

The data have been presented as mean ± SD

*P*-value: between-group comparison; independent sample t-test

^*^reflect significancy, WC waist circumference, *BMI* body mass index, *METs* metabolic equivalents

**Table 2:** Energy and macronutrients intake in the intervention and control groups before and after the intervention

Variable	Flaxseed group (*n* = 21)	Control group (n = 20)	*P*_1_-value
Energy (kcal)			
Before	1935.27 ± 488.7	1679.99 ± 316	0.093
After	1868.52 ± 462.86	1700.22 ± 313.41	0.168
P_2_-value	0.097	0.582	
Carbohydrate (g)			
Before	272.37 ± 68.83	234.89 ± 59.27	0.131
After	249.29 ± 70.95	242.60 ± 49.03	0.720
P_2_-value	0.006	0.274	
Protein (g)			
Before	84.39 ± 75.93	72.99 ± 22.86	0.179
After	60.93 ± 14.96	62.09 ± 14.69	0.073
P_2_-value	0.179	0.705	
Fat (g)			
Before	62.72 ± 18.57	54.46 ± 11.94	0.097
After	63.27 ± 19.68	56.65 ± 13.83	0.208
P_2_-value	0.865	0.378	
PUFA (g)			
Before	16.33 ± 6.03	14.73 ± 3.91	0.338
After	16.46 ± 5.32	15.19 ± 2.91	0.343
P_2_-value	0.849	0.471	
MUFA (g)			
Before	23.24 ± 6.57	19.84 ± 4.7	0.054
After	23.88 ± 6.62	20.53 ± 5.93	0.088
P_2_-value	0.620	0.552	
SFA (g)			
Before	17.7 ± 9.15	15.48 ± 5.6	0.397
After	16.89 ± 7.58	16.14 ± 6.38	0.725
P_2_-value	0.591	0.499	

SFA, saturated fatty acid; PUFA, polyunsaturated fatty acid; MUFA, monounsaturated fatty acids. The data have been expressed as mean ± SD. P1: between-group comparison of the variables; independent sample t-test (for energy and dietary protein, fat, PUFA, MUFA, and SFA) and Mann–Whitney U test (for dietary carbohydrate). P2: within-group comparison of the variables; paired sample t-test (for energy and dietary protein, fat, PUFA, MUFA, and SFA intake) and Wilcoxon signed-rank test (for dietary carbohydrate)

The mean anthropometric indices have been presented in Table 3. After 12 weeks of intervention, no significant difference was found between the two groups with respect to body weight, waist circumference, and BMI (*P* > 0.05). In both groups, a significant reduction was observed in body weight and BMI at the end of the study (*P* < 0.05). Although waist circumference significantly decreased in the flaxseed group (*P* = 0.001), no significant changes were observed in the control group (*P* = 0.467). As shown in Table 3, flaxseed powder consumption resulted in a significant decrease in serum insulin concentration (*p* = 0.003), HOMA-IR (*P* = 0.002), TG (*P* = 0.003), hs-CRP (*P* = 0.023), IL-6 (*P* = 0.047), and leptin (*P* = 0.016) and a significant increase in QUICKI (*P* = 0.001), HDL-C (*P* = 0.008), and adiponectin (*P* = 0.03) compared to the control group.

**Table 3:** The effects of flaxseed supplementation on serum levels of anthropometric indices and metabolic parameters in patients with polycystic ovary syndrome at baseline and after the intervention

Variables		Flaxseed group (*n* = 21)	Control group (*n* = 20)	Between-group comparison	
				P_1_	P_2_
Weight (kg)	Baseline	73.90 ± 15.20	76.8 ± 16.86	0.547	0.465
	End	72.99 ± 15.32	77.7 ± 16.73	0.329	0.006
	P_3_	0.031	0.014	–	–
Waist circumference (cm)	Baseline	88.71 ± 10.99	91.68 ± 13.99	0.433	0.260
	End	86.88 ± 11.34	93.47 ± 11.27	0.078	0.016
	P_3_	0.001	0.467	–	–
BMI (kg/m^2^)	Baseline	28.38 ± 5.15	28.94 ± 5.63	.729	0.537
	End	27.99 ± 5.13	29.31 ± 5.59	0.414	0.003
	P_3_	0.023	0.012	–	–
FBS (mg/dl)	Baseline	101.86 ± 12.08	101.77 ± 10.01	0.977	0.887
	End	94.25 ± 9.78	101 ± 11.31	0.056	0.021
	P_3_	0.151	0.606	–	–
Insulin (μIU/mL)	Baseline	12.17 ± 4.5	12.28 ± 3.35	0.136	0.647
	End	9.79 ± 3.78	13.69 ± 5.25	0.003	0.013
	P_3_	0.026	0.352	–	–
HOMA-IR	Baseline	3.04 ± 1.19	3.08 ± 0.89	0.658	0.566
	End	2.32 ± 1.06	3.35 ± 1.14	0.002	0.009
	P_3_	0.017	0.652	–	–
QUICKI	Baseline	0.32 ± 0.01	0.32 ± 0.01	0.673	0.716
	End	0.34 ± 0.02	0.32 ± 0.02	0.001	0.006
	P_3_	0.034	0.748	–	–
Total cholesterol (mg/dL)	Baseline	147.86 ± 33.32	153.16 ± 25.62	0.387	0.395
	End	140.25 ± 29.21	143.27 ± 22.72	0.727	0.871
	P_3_	0.301	0.118	–	–
LDL-cholesterol (mg/dL)	Baseline	84.09 ± 35.51	88.82 ± 41.86	0.684	0.844
	End	71.85 ± 32.21	78.03 ± 20.1	0.488	0.665
	P_3_	0.147	0.252	–	–
HDL-cholesterol (mg/dL)	Baseline	34.26 ± 11.75	37 ± 10.67	0.418	0.135
	End	43.3 ± 10.88	34.61 ± 7.5	0.008	P<
	P_3_	P < 0.001	0.285	–	0.001
Triglycerides (mg/dL)	Baseline	147.56 ± 21.59	156.77 ± 30.85	0.255	0.201
	End	125.5 ± 28.45	153.16 ± 25.62	0.003	0.007
	P_3_	P < 0.001	0.828	–	–
Total testosterone (ng/mL)	Baseline	0.93 ± 0.26	0.94 ± 0.25	0.666	0.994
	End	0.95 ± 0.25	0.96 ± 0.24	0.661	0.863
	P_3_	0.823	0.349	–	–
SHBG (nmol/L)	Baseline	45.02 ± 31.52	43.02 ± 12.57	0.286	0.610
	End	55.9 ± 9.69	49.05 ± 13.4	0.152	0.052
	P_3_	0.247	0.154	–	–
FAI	Baseline	2.49 ± 1.32	2.33 ± 1.02	0.219	0.319
	End	1.75 ± 0.55	2.21 ± 1.16	0.135	0.068
	P_3_	0.015	0.372	–	–
mF-G scores	Baseline	12.52 ± 7.53	11.04 ± 4.61	0.539	0.509
	End	11.43 ± 7.2	10.68 ± 4.2	0.879	0.726
	P_3_	P < 0.001	0.179	–	–
hs-CRP (mg/L)	Baseline	3.86 ± 1.53	3.81 ± 1.63	0.912	0.581
	End	2.87 ± 1.3	3.78 ± 1.03	0.023	0.021
	P_3_	0.02	0.596	–	–
IL-6 (pg/mL)	Baseline	4.54 ± 1.45	5.07 ± 1.51	0.166	0.329
	End	5.05 ± 1.7	5.08 ± 2.07	0.047	0.235
	P_3_	0.232	0.158	–	–
Adiponectin (mg/mL)	Baseline	13.04 ± 3.36	14.56 ± 4.16	0.044	0.073
	End	17.36 ± 4.1	14.18 ± 4.54	0.03	0.042
	P_3_	0.002	0.793	–	–
Leptin (ng/mL)	Baseline	70.18 ± 13.38	64.64 ± 21.67	0.633	0.329
	End	57.96 ± 10.19	70.97 ± 19	0.016	0.006
	P_3_	0.01	0.132	–	–

BMI, body mass index; FBS, fasting blood sugar; HOMA-IR, homeostatic model assessment of insulin resistance; QUICKI, quantitative insulin-sensitivity check index; LDL-C, low-density lipoprotein; HDL-C, high density lipoprotein; SHBG, sex hormone-binding globulin; FAI, free androgen index; mF-G score, modified Ferriman-Gallwey; hs-CRP, high sensitivity C-reactive protein; IL-6, interleukin-6

P1: between-group comparison of the variables; independent sample t-test (for FBS, TC, LDL, HDL, hs-CRP, adiponectin, leptin, and FAI) and Mann–Whitney U test (for other variables). P2: between-group comparison of the variables at baseline and after the intervention; analysis of covariance in the adjusted models (adjusted for age, BMI, dietary intake of energy, physical activity, and baseline biochemical variables). P3: within-group comparison of the variables; paired sample t-test (for FBS, TC, LDL, HDL, hs-CRP, adiponectin, leptin, and FAI) and Wilcoxon signed-rank test (for other variables)

The mean changes of all anthropometric and biochemical parameters have been presented in Table 4. The results indicated a significant decrease in body weight (P = 0.001), waist circumference (*P* = 0.007), BMI (*P* = 0.001), insulin concentration (*P* = 0.021), HOMA-IR (*P* = 0.034), TG (*P* = 0.013), and leptin (P = 0.007) and a significant increase in HDL-C (*P* < 0.001) and adiponectin (*P* = 0.017) in the flaxseed group compared to the control group.

**Table 4:** Mean changes (SD) of metabolic characteristics in the two study groups

Change from baseline	Flaxseed group (*n* = 21)	Control group (*n* = 20)	*P*_*1*_	*P*_*2*_
Weight (kg)	−0.9 ± 1.89	0.91 ± 1.58	0.001^a^	0.006^a^
Waist circumference (cm)	−1.95 ± 1.65	0.58 ± 3.32	0.007^a^	0.004^a^
BMI (kg/m^2^)	−0.38 ± 0.76	0.36 ± 0.62	0.001^a^	0.003^a^
FBS (mg/dL)	−6.95 ± 20.76	−2.22 ± 16.96	0.45	0.023^a^
Insulin (μIU/mL)	−2.75 ± 4.06	1.52 ± 6.74	0.021^a^	0.007^a^
HOMA-IR	−0.72 ± 1.13	0.26 ± 1.61	0.034^a^	0.009^a^
QUICKI	0.01 ± 0.02	− 0.004 ± 0.02	0.05	0.006^a^
Total cholesterol (mg/dL)	−8.3 ± 34.89	−14.11 ± 36.33	0.618	0.871
LDL-cholesterol (mg/dL)	−13.18 ± 39	−11.61 ± 41.54	0.905	0.665
HDL-cholesterol (mg/dL)	9.25 ± 6.59	−2.77 ± 10.68	P < 0.001^a^	P < 0.001^a^
Triglycerides (mg/dL)	−21.85 ± 22.06	− 0.27 ± 28.81	0.013^a^	0.011^a^
Total testosterone (ng/mL)	0.03 ± 0.35	0.07 ± 0.29	0.694	0.866
SHBG (nmol/L)	6.76 ± 30.36	6.35 ± 18.17	0.959	0.051
FAI	−0.73 ± 1.23	−0.13 ± 1.48	0.248	0.046^a^
mF-G scores	−1.09 ± 2.9	− 0.36 ± 3.11	0.121	0.254
hs-CRP (mg/L)	− 1.32 ± 2.08	−0.24 ± 1.93	0.108	0.011^a^
IL-6 (pg/mL)	0.62 ± 2.22	0.89 ± 2.58	0.731	0.237
Adiponectin (mg/mL)	4.31 ± 5.53	−0.39 ± 6.02	0.017^a^	0.041^a^
Leptin (ng/ml)	−9.48 ± 14.55	9.7 ± 25.93	0.007^a^	0.006^a^

The data have been expressed as mean ± SD. P1: between-group comparison of the mean changes of the studied variables; Mann–Whitney U (for waist circumference, insulin, QUICKI, and Ferriman-Gallwey score) and independent sample t-test (for other variables). P2: between-group comparison of the mean changes of the studied variables; analysis of covariance in the adjusted models (adjusted for age, BMI, dietary intake of energy, physical activity, and baseline biochemical variables)

^a^Statistically significant

The results revealed no significant differences between the two groups with regard to QUICKI and serum levels of fasting blood glucose, TC, LDL-C, inflammatory biomarkers, testosterone, SHBG and mF-G after the 12-week intervention.

In this study, an adjusted analysis was done on baseline biochemical variables, calorie intake, BMI, age, and METs. The results showed that flaxseed powder consumption significantly increased QUICKI (0.01 ± 0.02 vs. -0.004 ± 0.02, *P* = 0.006) and decreased fasting blood glucose (− 6.95 ± 20.76 vs. -2.22 ± 16.96 mg/dl, *P* = 0.023), hs-CRP (− 1.32 ± 2.08 vs. -0.24 ± 1.93 mg/l, *P* = 0.011), and FAI (− 0.73 ± 1.23 vs. -0.13 ± 1.48, *P* = 0.046). However, no significant change was found in other markers. The use of flaxseed could also significantly reduce body weight (− 0.9 ± 1.89 vs. 0.91 ± 1.58 kg, *P* = 0.006), waist circumference (− 1.95 ± 1.65 vs. 0.58 ± 3.32 cm, *P* = 0.004), and BMI (− 0.38 ± 0.76 vs. 0.36 ± 0.62 kg/m^2^, *P* = 0.003) (Table 4).

At baseline, 58.5% of the participants had menstrual irregularities (Table 5). After the intervention, however, menstrual regularity significantly increased in the flaxseed group compared to the control group (81% vs. 50%, *p* = 0.037) (Table 6).

**Table 5:** Menstrual status at the beginning of the study

Menstrual status	Flaxseed group (*n* = 21)	Control group (*n* = 20)	*P*-value
Regular [n (%)]	8 (38.1%)	9 (45%)	0.654
Irregular [n (%)]	13 (61.9%)	11 (55%)	

Chi-square test

**Table 6:** Menstrual status at the end of the study

Menstrual status	Flaxseed group (n = 21)	Control group (n = 20)	*P*-value
Regular [n (%)]	17 (81%)	10 (50%)	0.037
Irregular [n (%)]	4 (19%)	10 (50%)	

Chi-square test

## Discussion

Patients with PCOS are susceptible to many abnormalities in their biochemical factors, especially insulin metabolism [28]. In line with the present study results, Yari et al. indicated that flaxseed supplementation in patient with MetS for 12 weeks had beneficial effects on serum insulin level, HOMA-IR, and QUICKI. Similar findings were also obtained in other prior studies [29, 30]. This beneficial effect might be due to the higher amounts of lignan and fiber that can improve insulin sensitivity by reducing glucose uptake speed and insulin release [30]. Furthermore, some researchers have suggested that omega-3 fatty acids in flaxseed could increase adiponectin level, which has antiatherosclerotic, antidiabetic, and anti-inflammatory properties by improving insulin sensitivity. It has also been shown that adiponectin increased AMP-Activated Protein Kinase (AMPK) enzyme activity. AMPK activity was diminished in adipose tissue of very obese insulin-resistant people and was associated with IR and oxidative stress [31].

The present study results revealed no significant changes in testosterone and SHBG concentrations in the flaxseed group in comparison to the control group. In contrast, Nadjarzadeh et al. reported a significant reduction in serum total testosterone level without any significant effects on SHBG and FAI in patients with PCOS after supplementation with 3 g/day omega-3 for eight weeks. Ebrahimi et al. conducted a clinical trial and showed that supplementation with 1000 mg omega-3 fatty acids from flaxseed oil containing 400 mg ALA plus 400 IU vitamin E resulted in a significant improvement in serum total and free testosterone levels compared to the placebo. However, Andrea et al. reported that taking 4 g/day omega-3 for eight weeks did not change testosterone and SHBG levels significantly in patients with PCOS. The conflicting results of these studies point to the need for further research to examine the role of flaxseed consumption in androgens metabolism. After flaxseed supplementation in the current study, the percentage of regular menstruation was higher in the flaxseed group compared to the control group. This protective effect can act through several mechanisms. Higher intake of ALA is associated with increased concentration of enterolactone that can simulate SHBG synthesis and inhibit aromatase activity [32].

We showed that flaxseed powder consumption caused a significant improvement in the menstrual cycle regulation that was in line with previous findings [33, 34]. Most of the subjects in this trial had menstrual cycle disturbance. Similar to the results of this study, Nadjarzadeh et al. Reported that receiving omega-3 for 8 weeks could improve menstrual cycle periods [35].

In the current study, flaxseed supplementation reduced TG level and increased HDL level compared to the control group. In the same line, a study by Vargas et al. demonstrated that consumption of 3.5-g/d flaxseed oil significantly decreased TG concentration in patients with PCOS. Also, Taghizadeh et al. in a clinical study reported that taking 1000-mg omega-3 fatty acids from flaxseed oil plus 400-IU vitamin E led to a significant reduction in TG level and improvement in HDL level. Similar results were also obtained by Yari et al. Omega-3 fatty acids in flaxseed can change the transcription of hepatic genes involved in lipid metabolism, especially peroxisome proliferator-activated receptor-α and Sterol Regulatory Element Binding Protein-1 (SREBP1c) [36]. Indeed, it has been proved that omega-3 increased lipoprotein lipase activity and TG lipolysis [37].

The present study findings indicated that daily flaxseed supplementation decreased leptin level and improved adiponectin level. Consistently, Cassani et al. reported that flaxseed consumption in males with cardiovascular risk factors led to a significant increase in adiponectin concentration. Moreover, George et al. in a randomized clinical trial reported that taking 15 ml flaxseed oil for 12 weeks could not change adiponectin concentration significantly in 38 dyslipidemic male patients [38]. Omega-3 fatty acids in flaxseed can increase adiponectin production and release by up-regulation of PPAR-γ gene. ALA, the main fatty acid in flaxseed oil, is a ligand for PPAR-γ. PPAR-γ is responsible for the production of adiponectin [39]. Hence, flaxseed lignan acts as the PPAR-γ gene agonist and regulates adipogenesis-related transcription factors, such as adiponectin and leptin [40]. The mechanism whereby leptin mRNA expression is regulated by ALA has not been exactly discovered. Yet, it has been suggested that SREBP1c mRNA expression was regulated by ALA in the adipose tissue and an SREBP-like binding element was present in the leptin promoter that could be affected by PUFAs [41].

In the current study, flaxseed intake was associated with lower levels of hs-CRP. This finding was consistent with those of the previous studies [42, 43]. Secoisolariciresinol Diglucoside (SDG) is an active compound in flaxseed, which has anti-inflammatory effects [44]. Flaxseed also contains a high amount of soluble fiber fermented to short-chain fatty acids (acetate, propionate, and butyrate) by intestinal bacteria. Short-chain fatty acids, especially propionate, can down regulate inflammatory pathways in the body and reduce the levels of inflammatory agents, such as TNF-α, CRP, and IL-6 [45, 46].

The present study findings demonstrated that flaxseed supplementation for 12 weeks led to a significant reduction in body weight, waist circumference, and BMI in the women with PCOS. Obesity is one of the main causes of the onset of PCOS. Obesity does exacerbate the causes of PCOS, especially IR and dyslipidemia [47]. In the same line, Mohammadi-Sartang et al. in a systematic review and meta-analysis of 45 randomized placebo-controlled trials reported that flaxseed supplementation, especially in higher doses (≥ 30 g/day), could reduce body weight and waist circumference significantly. Previous studies suggested that SDG in flaxseed could reduce visceral (abdominal) fat by down regulation of mRNA levels of sterol regulatory element-binding proteins that are involved in TG synthesis [48]. SDG could also affect the circulation levels of fat oxidation related hormones, such as adiponectin, and increase fat oxidation in skeletal muscles [20]. Moreover, flaxseed is a rich source of fibers, specifically soluble fiber (25% soluble form). Dietary fiber, especially soluble fiber, could help weight loss by delayed gastric emptying and inducing the feeling of fullness [49].

The strengths of this study were its design as a controlled clinical trial and use of whole flaxseed instead of flaxseed oil or lignan. However, this study had a few limitations that need to be considered. The main limitation of the study was its open-labeled design resulted from the lack of a proper placebo. In addition, it was not possible to evaluate the ALA content of erythrocyte membranes, which could have been used as a marker of adherence to flaxseed supplementation.

## Conclusion

The present study was the first report of the effectts of flaxseed powder on glycemic indices, lipid profile, inflammatory factors, adipocytokines, and androgen profile in women with PCOS. The results indicated that flaxseed powder supplementation (30 g/day) among patients with PCOS for 12 weeks had beneficial effects on insulin metabolism, body composition related factors, hs-CRP, TG, HDL, adiponectin, leptin, and mF-G score. In conclusion, this randomized controlled clinical trial found some evidence that flaxseed supplementation in patients with PCOS could improve some biochemical and anthropometric markers, at least partially, through amelioration of dyslipidemia, obesity, IR, and inflammation. Yet, further studies are needed to identify the exact mechanisms of these beneficial effects and to determine the safe dosages.

As a summary, this randomized controlled clinical trial is one of the first studies to document the beneficial effects of 12-week supplementation with 30 g/day flaxseed in polycystic ovary syndrome.

## Data Availability

Data that support the findings of this study are available from the corresponding author upon reasonable request.

## Abbreviations

ALA: α-Linolenic Acid
AMPK: AMP-Activated Protein Kinase
BMI: Body Mass Index
CRP: *c-reactive protein*
CV: Coefficient of Variation
CVD: Cardiovascular Disease
FAI: Free Androgen Index
FBS: fasting blood sugar
HDL-C: High-Density Lipoprotein Cholesterol
HOMA-IR: Homeostatic Model Assessment of Insulin Resistance
hs-CRP: high-sensitivity C-Reactive Protein
IL-6: Interleukin 6
IPAQ: International Physical Activity Questionnaire
IR: Insulin Resistance
LDL-C: Low-Density Lipoprotein Cholesterol
LM: Lifestyle Modification
METs: Metabolic Equivalents
MetS: Metabolic Syndrome
mF-G-score: *Modified* Ferriman–Gallwey
MUFA: Monounsaturated fatty acids
PCOS: Polycystic Ovary Syndrome
PPAR-γ: *peroxisome proliferator-activated receptor-*γ
PUFA: Polyunsaturated Fatty Acid
QUICKI: Quantitative Insulin-Sensitivity Check Index
SD: Standard Deviation
SDG: Secoisolariciresinol diglycoside
SFA: Saturated fatty acid
SHBG: Sex Hormone-Binding Globulin
SREBP1c: Sterol Regulatory Element Binding Protein-1
TC: Total Cholesterol
TG: Triglycerides
TNF-α: *Tumor necrosis factor alpha*
WC: Waist Circumference
WHO: World Health Organization
